# Alcohol use patterns and disorders among individuals with personality disorders in the Sao Paulo Metropolitan Area

**DOI:** 10.1371/journal.pone.0248403

**Published:** 2021-03-23

**Authors:** Carolina Hanna Chaim, Geilson Lima Santana, Paula de Vries Albertin, Camila Magalhães Silveira, Erica Rosanna Siu, Maria Carmen Viana, Wang Yuan Pang, Laura Helena Andrade

**Affiliations:** 1 Section of Psychiatric Epidemiology, Institute & Department of Psychiatry (LIM-23), University of Sao Paulo Medical School, Sao Paulo, SP, Brazil; 2 Department of Social Medicine, Federal University of Espirito Santo, Vitoria, ES, Brazil; University of the Witwatersrand, SOUTH AFRICA

## Abstract

**Introduction:**

Alcohol Use Disorders are frequently comorbid with personality disorders. However, the heterogeneity of the prevalence estimates is high, and most data come from high income countries. Our aim is to estimate the prevalence and association between alcohol use outcomes and the three DSM-5 clusters of personality disorders in a representative sample of the São Paulo Metropolitan Area.

**Materials and methods:**

A representative household sample of 2,942 adults was interviewed using the WHO Composite International Diagnostic Interview and the International Personality Disorder Examination Screening Questionnaire. Lifetime PD diagnoses were multiply imputed, and AUD diagnoses were obtained using DSM-5 criteria. We conducted cross-tabulations and logistic regression to estimate the associations between AUDs and PDs.

**Results and discussion:**

Our study did not find significant associations of PDs with heavy drinking patterns or mild AUD. Cluster B PD respondents tended to show the highest conditional prevalence estimates of most alcohol use patterns and AUD, including its severity subtypes. When alcohol outcomes were regressed on all PD Clusters simultaneously, with adjustment for sex and age, only cluster B was significantly associated with past-year alcohol use (OR 3.0), regular drinking (OR 3.2), and AUDs (OR 8.5), especially moderate and severe cases of alcohol use disorders (OR 9.7 and 16.6, respectively). These associations between Cluster B PDs and these alcohol outcomes were shown to be independent of other PD Clusters and individuals´ sex and age.

**Conclusion:**

The main finding of our study is that AUDs are highly comorbid with PDs. The presence of Cluster B PDs significantly increases the odds of alcohol consumption and disorders and of more severe forms of AUDs. Considering the local context of poor treatment provision, more specific prevention and intervention strategies should be directed to this population.

## Introduction

Alcohol use is currently a widespread phenomenon [1], encompassing a spectrum of diverse drinking patterns. The most severe alcohol use-associated outcomes are alcohol use disorder (AUDs) [2], a pattern of alcohol consumption that results in overall physical, mental and/or social health impairment [3]. AUDs are frequently comorbid with personality disorders (PDs) [4]. However, the heterogeneity of the prevalence estimates are high [5], and the majority of data come from high income countries [4].

PDs are defined as an enduring pattern of inner experience and behavior that deviates markedly from the expectation of the individual’s culture, according to the Diagnostic and Statistical Manual of Mental Disorders, Fifth Edition (DSM-5). PDs are pervasive and inflexible, begin in adolescence or early adulthood, are stable over time, and lead to distress or impairment [3]. Worldwide PD prevalence in the general population is estimated as 7.8% [6].

The ten specific PD categories described by the DSM-5 are further grouped into three clusters based on phenotypical similarities. Cluster A, the “odd or eccentric” group, includes paranoid, schizoid and schizotypal PDs. Cluster B, the “dramatic, emotional and/or erratic” group, includes antisocial, borderline, histrionic and narcissistic PDs. Cluster C, the “anxious or fearful” group, includes avoidant, dependent and obsessive-compulsive PDs [7].

The National Epidemiological Survey on Alcohol and Related Conditions (NESARC) found that individuals with any PD are five times more prone to have alcohol dependence, compared to individuals with no PD [8]. In addition, approximately 42% of those with any PD also presented lifetime alcohol dependence in this representative nonclinical sample. In contrast, prevalence in clinical samples were found to be as high as 62.2% [9].

Few studies sought to clarify patterns of comorbidities associated with the three Clusters separately [10, 11], and the most consistent finding is the association with Cluster B [12–14]. For instance, in the National Comorbidity Survey Replication, the prevalence of 12-month AUD was 5.8% among Cluster A subjects; 26.7% for Cluster B; and 5.4% for Cluster C. However, the only statistically significant association was found for Cluster B, with an odds ratio of 10.3 (95% CI 5.7–18.7) [11]. In general, there is a scarcity of data on the association of alcohol outcomes with PD Clusters A and C.

Regarding the ten specific categories of PDs, the NESARC study found highly prevalent and statistically significant associations of AUDs with all specific PD categories, especially for Cluster B PDs (antisocial, 49.19%, OR 7.76; borderline, 47.41%, OR 5.37; histrionic 49.79%, OR 6.98; narcissistic, 39.03%, OR 3.61); but also for Cluster A PDs (paranoid, 38.27%, OR 4.53; schizoid, 37.82%, OR 4.30; schizotypal, 42.38%, OR 4.13) and Cluster C (avoidant, 34.91%, OR 3.94; dependent, 28.04%, OR 2,73; obsessive-compulsive, 31.85%, OR 3.38) [8].

Three main theoretical pathways were proposed to explain the dual diagnosis composed by PDs and AUDs, which are not necessarily exclusive. The first theorizes that PD features could primarily contribute to AUD development since alcohol is used repeatedly to manage emotional states (self-medication hypothesis) [15, 16]. This rationale is supported by data suggesting that having any PD is a predictor of transitioning from substance use to dependence [17] and that the comorbid mental disorder typically starts at an earlier age than the AUD [18]. The second pathway proposed is that alcohol use problems precede the onset of PD and contribute to its development. Adolescence is a crucial period for personality development and consolidation of protective personality traits, such as high constraint and positive emotionality, and frequent alcohol use could interfere with important adaptations [19–21]. Third, epigenetic might contribute to the complex mechanisms behind PD and AUD comorbidity, since there are common associated genotypes and shared environmental factors such as childhood adversities [22, 23]. Traits of neuroticism (or negative emotionality) and disinhibition would be common to PDs and AUDs, argumenting in favour of the shared vulnerability model [24, 25].

Some studies have explored the role of specific personality traits and PD Clusters characteristics as risk factors for AUDs [26]. Cluster A individuals may suffer from high levels of neuroticism, especially schizotypal subjects, and alcohol consumption may be a means of reducing stress [26]. Behavioral disinhibition, low harm avoidance and impulsivity are common Cluster B traits associated with AUDs, as well as neuroticism and negative affectivity [27] and narcissism [28]. Furthermore, high novelty and sensation seeking may be present in histrionic and narcissistic PDs, predisposing to alcohol and other substance use and disorders [26]. Finally, Cluster C PD individuals suffer from excessive fear and anxiety. Along with the self-medication theory, it is hypothesized that consumption of alcohol and other substances is aimed at counteracting feelings of isolation and a lack of social relations among those individuals [29].

In the present study, we aim to explore the associations between any lifetime PD and Clusters of PDs with diverse patterns of alcohol use along a spectrum comprised of increasing drinking levels and frequencies, and AUD and its severity levels in a representative population from a Brazilian megacity, which is located in southeastern Brazil, with approximately 20 million inhabitants [30], corresponding to more than 10% of the Brazilian population [31].

## Materials and methods

### Sample

This study is part of the “São Paulo Megacity Mental Health Survey (SPMHS) [32, 33], the Brazilian branch of the “World Mental Health Survey Initiative” (WMH), coordinated by the World Health Organization and Harvard University [34]. The SPMHS is a cross-sectional representative survey of household residents aged 18 years or older in the SPMA, a region that comprises the city of Sao Paulo and 38 surrounding municipalities. Data were collected between 2005 and 2007, and during that period, more than 11 million inhabitants were adults in this region [35]. Respondents were selected by means of a stratified, multistage area probability sample of households. To achieve the planed sampling of 5,000 households, 7,700 households were initially targeted, allowing a 35% non-response rate. Two strata were defined (the city of Sao Paulo and the 38 surrounding municipalities). Each municipality contributed to the total sample size according to its population size, and six selection stages were used to recruit the sample in these two geographic strata. In all strata, the primary sampling units (PSUs) were the year 2000 census count areas defined by the Brazilian Institute of Geography and Statistics (IBGE, in the Brazilian abbreviation) [35].

The first stage defined 134 PSUs. In the second stage, 21,158 IBGE Census Units (CUs) were delineated. A CU is the smallest unit with available census data and comprise 200 to 500 households. In the third stage, CUs were clustered within each PSU. To achieve the goal of sampling 5,000 households, 1,540 were selected. In the fourth stage, one CU was randomly selected within each PSU. In the fifth stage, one block from each CU was randomly selected from a map of the area, where all blocks were numbered. All households from the selected blocks were recorded with street names and numbers. Households were then randomly selected.

In the last stage of sampling, in each household, the interviewer obtained a list of all residents, with information on age, sex, and family relationship to the informant. The eligible respondents were identified, i.e., those who were 18 years or older, Portuguese-speaking and without any disability or handicap that would impair their ability to participate in the study. This list was then sorted by gender and inverse order of age. One resident was then randomly selected by means of a Kish grid, a probabilistic method for selecting household respondents from a table of random numbers [36]. In addition, in a random 20% sample of households where the selected respondent was married or living as married, the spouse was identified and selected for interview. The survey had a global response rate of 81.3%, resulting in a total of 5,037 subjects that were evaluated.

Detailed descriptions of the sampling procedures and corresponding steps are presented elsewhere [32].

The São Paulo Megacity Mental Health Survey was approved by the Ethical and Research Committee of the University of Sao Paulo Medical School (Process 792/03). Respondents were interviewed only after written informed consent was obtained and confidentiality was assured.

### Assessment

Face-to-face interviews were conducted by trained professional personnel using the World Mental Health version of the Composite International Diagnostic Interview (WMH-CIDI) [37]. To avoid respondents´ burden, the WMH-CIDI interview was divided in two parts. Part 1 was administered to all respondents (n = 5,037) and assessed ‘core’ psychiatric disorders (mood, anxiety, substance, and impulse control disorders), sociodemographic information, daily functioning, and physical morbidity. Immediately after completing Part 1 modules, all respondents who met lifetime criteria for any core disorder, plus a 25% random sample of non-cases were assessed with Part 2 modules, which included screening questions for personality disorders, other mental disorders, as well as risk factors, consequences and other correlates of psychopathology. Part II respondents (2,942 subjects) are the focus of the current report. As detailed in the “Data Analysis” section, a set of weights were used to allow both Part 1 and 2 samples to be representative of the general population and, particularly in Part 2 sample, to prevent the effect of an oversampling of mental disorders.

### Measures

#### Predictors

Personality Disorders Clusters were assessed with 33 screening questions from the International Personality Disorder Examination (IPDE) [38, 39], following the procedures of multiple imputation adopted by the WMH [40].

These 33 items were shown to be significant predictors of one or more of the PD Clusters (A, B and C) or the overall diagnosis of any personality disorder assessed by a clinician-administered IPDE [41, 42]. Responses to these questions were combined to generate diagnoses based on a calibration study with a probability subsample of Part II respondents (n = 214) of the US National Comorbidity Survey Replication, oversampling those who screened positive to PDs [11]. These respondents were assessed in clinical reappraisal interviews with the complete IPDE by a veteran and well-trained clinician, blind to the screening responses. The next step was to link screening responses with the IPDE clinical diagnoses of Clusters A, B and C and any PD. Predicted probabilities of these four diagnoses were assigned to each respondent based on responses to the screening questions using results of stepwise logistic regression in the clinical reappraisal sample. Prediction accuracy in the calibration sample was considered excellent in all equations, with area under the receiver operating characteristic curve (AUC) of 0.94 for Cluster A, 0.92 for Cluster B, 0.90 for Cluster C and 0.88 for any PD [11].

These prediction equations were used to estimate the diagnoses of Clusters A, B, C and any PD by means of multiple imputation in participating countries of the WMH, including the SPMHS. Multiple imputation procedures are described in more details in the “Data Analysis” section.

Clusters A, B, C, and any PD are binary variables coded as 0 (absence) or 1 (presence of the PD diagnosis).

#### Alcohol use outcomes

Alcohol use was measured by the alcohol module of WMH-CIDI [37]. Participants answered questions regarding alcohol use, drinking patterns, and related disturbances. Five drinking patterns were included in this analysis.

Those who consumed at least one drink in the previous 12 months were termed ‘past-year users’.

Those who consumed at least 12 drinks in the previous 12 months formed a heterogeneous subgroup distinguished with the term ‘regular user’ [2, 43, 44]. Within this subgroup, three mutually exclusive subgroups were formed [2]: ‘heavy drinkers of low frequency’ who have consumed five or more drinks in a row for men, and four or more drinks in a row for women, but no more often than two times per month; ‘heavy drinkers of high frequency’ for whom heavy drinking occurred at least three times per month; and ‘non-heavy drinkers’.

DSM-5 AUD diagnoses were created using a set of questions derived from the DSM-IV and the 10^th^ Edition of the International Classification of Diseases (ICD-10) alcohol abuse/dependence criteria embedded in the WMH-CIDI [45]. Matching the DSM-5 criteria, 11 dichotomous variables were generated, and positive cases had to endorse at least two criteria (Tolerance, Withdrawal, Larger/Longer, Quit/Control, Time Spent, Activities Given Up, Physical/Psychological, Neglect Roles, Social/Interpersonal, Hazardous Use and Craving). In addition, DSM-5 AUD cases were classified according to three levels of severity based on the number of criteria endorsed: mild (2 or 3 criteria); moderate (4 or 5 criteria); or severe (6 or more criteria). In this study, we examined DSM-5 AUD among regular alcohol users.

All alcohol use patterns, and the DSM-5 AUD diagnosis are binary variables coded as 0 (absence) or 1 (presence of the alcohol use pattern of AUD diagnosis). A second variable related to DSM-5 AUD is a categorical variable with four categories: absence of diagnosis (reference category); mild AUD (2 or 3 criteria); moderate (4 or 5 criteria); and severe AUD (6 or more criteria).

#### Correlates

Sex (coded as female or male) and age (a continuous variable varying from 18 to 93 years old) were included in the statistical models as control variables, as detailed below.

### Data analysis

#### Multiple imputation of Clusters A, B and C and of any PD

Analyses were performed using Part II sample (n = 2,942). Instead of using screening questions to generate diagnoses of clusters A, B or C or any PD, we adopted an alternative procedure, based on the US National Comorbidity Survey Replication (NCS-R), the prototypical study of the WMH project [46–48]. To generate the diagnoses, we used multiple imputation (MI), a technique that makes it possible to include the uncertainty of the imputation in the results, providing valid inferences of missing values [49]. MI provides statistically valid inferences in the context of values missing completely at random, as is the case of planned missingness [46–48], a strategy adopted by all WMH participant sites, including the Sao Paulo Megacity survey.

Hence, we imputed PD diagnoses from IPDE screening questions using the prediction equations obtained in the US clinical reappraisal study [40]. In the US clinical reappraisal study ten prediction equations were created for each of the four diagnoses; those predictors included personality screening items, sociodemographic variables and questions related to other mental disorders. Ten predicted probabilities for each PDs diagnose were assigned to each respondent and were used to create ten multiple imputation datasets. Prediction accuracy in the calibration sample was considered excellent for all the equations [11].

The MI is a three steps process: (i) imputation phase, where ten datasets were generated for each diagnosis (clusters A, B, C and any PD); (ii) analyses were carried out separately on each dataset, resulting in ten sets of parameters estimates; and (iii) the resulting ten sets of estimates were averaged to obtain a best estimate of the parameter, and coefficients and standard errors were adjusted for the variability between imputations according to Rubin’s rules (more details can be consulted at [43, 50].

#### Complex survey analysis

All analyses were conducted using Stata 15 [51]. Due to the complex sample design, imputed parameters were estimated using the “*mi estimate*: *svy*:*”* command [52], accounting for stratification, clustering and weighting. Concisely, data were weighted to adjust for the probabilities of selection and non-response in households on part II of the interview, and to adjust for residual discrepancies between sample and population distributions on a range of socio-demographic variables. In this way, our sample is representative of the adult general population resident in the Sao Paulo Metropolitan Area.

#### Stages of data analysis

First, we described our sample according to age, sex, personality disorders, alcohol use patterns and AUD. To focus our analysis on recently active drinkers our estimates are “conditional”, since we estimated regular alcohol use only among past-year drinkers, and heavy drinking and AUD only among regular users. “All other (subjects) are assumed to be effectively not at risk for being an active heavy drinker or for qualifying as a case of a DSM-5 alcohol disorder in the past year” [2].

Second step, cross-tabulations were used to estimate, among individuals with PD, the overall prevalence of recently active (past-year) drinking, as well as “conditional prevalence” estimates of regular use, heavy drinking and AUD and corresponding levels of severity.

Third step, we assessed the association of any PD with past-year alcohol use, regular use, heavy drinking, and AUD in a series of six logistic regressions, adjusted for sex and age. Then we evaluated the association of any PD with AUD severity by a multinomial logistic regression adjusted for sex and age.

In the fourth step, we assessed the association of PD Clusters with past-year alcohol use, regular use, heavy drinking, and AUD in a series of six logistic regressions. For that, we simultaneously included all three PD Clusters and adjusted for sex and age. After that, we examined the association of PD Clusters with AUD severity by a multinomial logistic regression, once again simultaneously including all three PD Clusters and adjusting for sex and age. Confidence intervals (CIs) of the odds ratios (ORs) were estimated using the Taylor series method. Statistical significance was based on two-sided tests evaluated at the 0.05 level Multicollinearity was verified with the variance inflation factor (VIF) statistics.

## Results

### Sample characteristics according to gender, age, personality disorders and alcohol use patterns and disorder

The weighted mean age of participants was 39.1 years old. The study sample had a balanced distribution in relation to participants’ sex. As seen on Table 1, 52.8% were women and 47.2% were men. The weighted prevalence of any personality disorders was 6.8%, and the proportion of individuals with a Clusters A, B or C PD were, respectively, 4.3%, 2.7% and 4.6% [53].

**Table 1:** Weighted mean of age and weighted prevalence estimates of sex, lifetime personality disorders and past-year alcohol use, drinking patterns and alcohol-use disorder in the São Paulo Metropolitan Area, Brazil (N = 2,942).

Sociodemographic correlates	Weighted mean estimate (min-max)
**Age (continuous)**	39.1 years old (18–93 years old)
	**Prevalence estimate (standard error)**
**Sex**	
**Female**	52.8% (1.3)
**Male**	47.2% (1.3)
**Personality disorders** ^**a**^	
**Any**	6.8% (1.0)
**Cluster A**	4.3% (0.7)
**Cluster B**	2.7% (0.5)
**Cluster C**	4.6% (0.7)
**Past-year alcohol use** ^**b**^	45.5% (1.5)
**Regular use**^**1**^	71.3% (2.1)
**Non-heavy drinking**	70.3% (2.4)
**Heavy drinking of lower frequency**	9.5% (1.1)
**Heavy drinking of higher frequency**	20.2% (2.0)
**DSM-5 Alcohol use disorder**^**2**^	23.1% (2.0)
**Mild**	10.6% (2.0)
**Moderate**	5.6% (0.8)
**Severe**	7.0% (1.0)

Part II weight.

^**a**^ Already published in (53).

^b^ According to the methodology described by (2).

^1^ Among past-year users.

^2^ Among regular users.

Almost half (45.5%) of the participants reported alcohol consumption in the previous years, and among them, 71.3% were regular users. One fifth (20.2%) of these regular users had a heavy drinking of higher frequency, and 23.1%, an alcohol use disorder, distributed into mild (10.6%), moderate (5.6%) and severe (7.0%) cases.

### Conditional prevalence estimates of alcohol use patterns and AUD among individuals with PDs

According to Table 2, more than half (55.2%) of individuals with any PD reported using alcohol the previous years, and 80.6% of them were regular users. Most of these regular users were not heavy drinkers (62.0%), and almost 30% showed a pattern of heavy drinking of higher frequency.

**Table 2:** Weighted conditional prevalence estimates of past-year alcohol use, drinking patterns and alcohol-use disorder among subjects with personality disorders in the São Paulo Metropolitan Area, Brazil (N = 2,942).

Personality disorders	Past-year use	Regular use ^1^	Among regular drinkers				Severity of DSM-5 AUD ^2^		
			Non-heavy drinking	Heavy drinking of lower frequency	Heavy drinking of higher frequency	DSM-5 AUD	Mild	Moderate	Severe
	% (SE)	% (SE)	% (SE)	% (SE)	% (SE)	% (SE)	% (SE)	% (SE)	% (SE)
**Any**	55.2% (7.2)	80.6% (5.4)	62.0% (8.4)	8.3% (6.2)	29.7% (8.5)	47.3% (9.7)	11.4% (5.3)	12.9% (5.0)	23.0% (6.3)
**Cluster A**	64.9% (8.4)	80.4% (7.0)	56.5% (12.4)	10.7% (9.3)	32.7% (11.7)	42.3% (11.4)	8.6% (4.9)	14.1% (6.9)	19.5% (7.0)
**Cluster B**	72.4% (7.0)	89.2% (4.6)	52.7% (13.4)	11.0% (7.4)	36.3% (11.6)	69.8% (14.2)	12.9% (5.2)	19.8% (8.6)	37.1% (10.3)
**Cluster C**	48.0% (6.8)	79.3% (7.5)	70.6% (11.5)	5.8% (6.5)	23.6% (10.8)	46.1% (10.7)	12.5% (6.3)	11.2% (6.1)	22.4% (7.4)
**None**	44.7% (1.7)	70.5% (2.3)	71.3% (2.5)	9.3% (1.2)	19.4% (2.1)	20.6% (2.0)	10.6% (1.4)	4.8% (0.9)	5.2% (0.9)

Part II weight.

^1^ Among past-year users.

SE: Standard error.

PD: personality disorder.

DSM-5 AUD: Alcohol use disorder according to DSM-5.

Almost half (47.3%) of PD regular drinkers had an AUD. Among these individuals, the PD prevalence tended to be higher among those with more severe AUD (varying from 11.4% to 23.0%). This tendency could not be tested due to limitations of the multiple imputation procedure, which does not allow chi-square test.

The prevalence of past-year alcohol use among cluster A PD subjects was 64.9%, and 80.4% of these were regular users. Most cluster A regular drinkers (56.5%) did not have a heavy drinking pattern, and 42.3% had an AUD. Once again there was a tendency of higher conditional prevalence estimates the greater the AUD severity (varying from 8.6% to 19.5%). This tendency also could not be tested due to limitations of the multiple imputation procedure, which does not allow chi-square test.

Cluster B PD respondents tended to show the highest conditional prevalence estimates of most alcohol use patterns and AUD, including its severity subtypes. The estimate of past-year alcohol consumption was 72.4%, and most of these (89.2%) drank regularly. Among regular drinkers, the conditional prevalence of heavy drinking of low- and of high-frequency were 11.0% and 36.3%, respectively, and almost 70% of regular drinkers had an AUD. Once again, there was a tendency of an increasing prevalence the higher the AUD severity (12.9% for mild, 19.8% for moderate, and 37.1% for severe AUD), which could not be tested for the same reason already described.

Compared to other PDs, subjects with a cluster C PD showed a lower estimate of past-year alcohol use (48.0%), and among the regular users, a lower estimate of heavy drinking (29.4%). Almost half (46.1%) of cluster C regular drinkers had an AUD (12.5% for mild, 11.2% for moderate and 22.4% for severe AUD).

Individuals without PD had lower estimates of past-year use (44.7%). The conditional prevalence of regular drinking among past-year users was high (70.5%), although lower than the estimates of subjects with PDs. Another contrast to PD subjects is that most of these regular alcohol users do not heavy drink (71.3%), and had lower estimate of AUD (20.6%). While severe AUD had the greatest prevalence estimates in individuals with personality disorders, mild AUD predominated in subjects without the comorbidity (10.6%), tending to reverse the aforementioned severity gradient.

### Associations of personality disorders with alcohol use patterns and AUD

Table 3 shows that, regardless of age and sex, any PD had a significant association with DSM-5 alcohol use disorder (OR 3.3), especially with moderate and severe cases (ORs 3.7 and 6.4, respectively).

**Table 3:** Associations of personality disorders with past-year alcohol use, drinking patterns and alcohol-use disorder in the São Paulo Metropolitan Area, Brazil (N = 2,942).

Personality disorders	Past-year use	Regular use ^1^	Among regular drinkers			DSM-5 AUD ^2^	Severity of DSM-5 AUD ^2^		
			Non-heavy drinking	Heavy drinking of lower frequency	Heavy drinking of higher frequency		Mild	Moderate	Severe
	OR (95% CI)	OR (95% CI)	OR (95% CI)	OR (95% CI)	OR (95% CI)	OR (95% CI)	OR (95% CI)	OR (95% CI)	OR (95% CI)
	*p*	*p*	*p*	*p*	*p*	*p*	*p*	*p*	*p*
**MODEL 1**									
**Any**	1.2 (0.6–2.5)	1.5 (0.7–3.3)	0.8 (0.3–1.7)	0.6 (0.1–3.8)	1.6 (0.6–4.2)	3.3 (1.3–8.4)	1.6 (0.4–6.2)	3.7 (1.1–12.9)	6.4 (2.2–18.6)
	0.560	0.249	0.483	0.598	0.336	0.014	0.516	0.037	0.002
**MODEL 2**									
**Cluster A**	1.3 (0.5–3.3)	1.1 (0.4–3.4)	0.8 (0.2–2.7)	0.7 (0.0–10.8)	1.4 (0.4–4.9)	1.5 (0.4–5.4)	0.8 (0.1–4.9)	2.1 (0.4–12.0)	2.0 (0.4–11.0)
	0.555	0.858	0.654	0.770	0.544	0.502	0.790	0.387	0.387
**Cluster B**	3.0 (1.4–6.7)	3.2 (1.1–9.4)	0.5 (0.1–2.4)	1.0 (0.1–6.5)	1.9 (0.6–6.4)	8.5 (1.7–42.7)	3.6 (0.7–17.8)	9.7 (1.0–90.5)	16.6 (2.9–96.2)
	0.009	0.031	0.376	0.988	0.258	0.014	0.105	0.047	0.005
**Cluster C**	1.0 (0.5–1.7)	1.4 (0.4–4.8)	1.1 (0.3–3.9)	0.6 (0.1–4.8)	1.0 (0.2–4.2)	2.2 (0.8–6.1)	1.6 (0.3–7.1)	2.1 (0.4–9.6)	3.4 (0.9–12.34)
	0.897	0.495	0.883	0.628	0.983	0.106	0.544	0.341	0.060

Part II weight.

MODEL 1: Adjusted for sex and age.

MODEL 2: Adjusted for sex, age, and other PD Clusters.

PD: personality disorders; DSM-5 AUD: alcohol use disorder according to DSM-5.

^1^ Among past-year users.

^2^ Among regular users.

OR: odds ratio; 95% CI– 95% confidence interval.

In the second models, where alcohol outcomes were regressed on all PD Clusters simultaneously, with adjust for sex and age, only cluster B was significantly associated with past-year alcohol use (OR 3.0), regular drinking (OR 3.2), and AUD (OR 8.5), especially moderate and severe cases of alcohol use disorder (OR 9.7 and 16.6, respectively). These associations between Cluster B PD and these alcohol outcomes were shown to be independent of other PD clusters and individuals´ sex and age.

Our study did not find significant associations of PDs with heavy drinking patterns or mild AUD.

According to post-estimation VIF statistics, multicollinearity was not detected.

## Discussion

The main finding of our study is that AUD are highly comorbid with PDs. The presence of PD significantly increases the odds of AUD and of more severe forms of AUD. Noteworthy, PDs did not increase the odds of past-year alcohol use, regular drinking or heavy drinking. One possible explanation is that alcohol consumption habits are widespread in the SPMA, even among those without a mental disorder [2]. Nonetheless, the contributing factors involved in the transition from non-problematic drinking to AUD should be a major concern in this population.

Previous literature adopting DSM-IV criteria of abuse and dependence has shown a strong association between PD and alcohol dependence. However, this phenomenon was not always true for alcohol abuse, even when adjusted for sociodemographic variables and other psychiatric disorders [17, 54]. This is consistent with our findings, since there was a clear tendency of association with severe AUD, but not completely for mild AUD.

In our study, about 70% of Cluster B PD individuals have AUD. Earlier available results are very heterogeneous and rates range from 11.9% to 66% [55]. In both alcohol dependence and cluster B PD, there is an impairment related to decision making and behavioral control, which was more evident in patients with alcohol dependence and cluster B PD comorbidity [12]. Cluster B PD individuals had odds almost nine times higher for an AUD when compared with those without cluster B PD. Most of the previous results on PD explored cluster B types of PD [14, 55, 56], which demonstrated higher rates of comorbidity with substance use disorders among all types of PD.

In our study, mild AUD was common in the general sample and also among those reporting PD. The results support the evidence that this diagnose may be too lenient, particularly among youth, in which the symptoms of tolerance and hazardous use could reflect no clinical significant harm [57]. On the other hand, the DSM-5 approach might classify a proportion of DSM-IV’s diagnostic orphans and this lower threshold could improve prevention strategies and availability of treatment [58] In fact, in one of our previous reports of the SPMHS, using latent class analyses, we found two symptomatic classes that represented well the dimensionality of the DSM-5 AUD criteria. Most of the individuals from the “use in larger amounts class” were diagnosed with mild [54.8%] or moderate [28.3%] DSM-5 AUD, while almost all individuals from the “high-moderate symptomatic class” were diagnosed as having severe DSM-5 AUD (96.7%). We considered that DSM-5 AUD criteria have the advantage of shedding light on risky drinkers included in the “use in larger amounts class,” allowing for preventive and brief interventions, which may target a large number of individuals [45].

Lack of access to health resource support for mental disorders is a reality in the SPMA, especially for patients with personality disorders. Only one in five cases of those with any PD had received treatment for emotional or substance use problems in the year previous to the interview [33, 53]. Santana and colleagues found that among individuals with PDs, treatment was predominantly associated with comorbid, rather than “pure” PDs. Comorbidity may result in greater severity of symptoms and, thus, increased treatment search and service use [53]. On the other hand, individuals with alcohol use problems and PD may have poorer outcomes and a lower perception of these problems [59]. PD is associated with early dropout of treatment for AUD, but when patients stay on treatment the outcomes are usually favorable, which reinforces the importance of recognizing PD [5]. In this way, the results from our study highlight the need of appropriate assessment of PDs in patients with AUD and, conversely, the need of an appropriate assessment of AUD in patients with PD.

The present study used a solid database generated from an observational research of a representative sample of the SPMA, and therefore its results can be generalized for this population. This is one of very few studies in developing countries assessing the prevalence and associations of alcohol use and AUD with PDs in a representative sample of the general population.

Our study has limitations. Our data do not allow studying specific categories of PDs, but only PD clusters and any PD, undermining more specific understanding. For instance, among cluster B disorders, borderline and antisocial types are the most studied and data on histrionic and narcissistic types are still scarce. Epidemiological data, however, suggest that PD type accounted for most of the heterogeneity in lifetime AUD prevalence [4]. In addition, research conducted by retrospective self-reporting involves the risk of recall bias. As multiple imputation is unbiased in estimating prevalence when applied to a single population [60], the possibility of bias implies that the imputation rules, which were based on clinical calibration in the USA, might not be accurate in the other WMH countries. To address this possibility, future cross-national epidemiological surveys need to go beyond the exclusive use of screening questions to administer full personality disorder clinical interviews in community samples in multiple countries and to carry out clinical reappraisal interviews in a substantial subsample in each country. Diagnoses were generated from IPDE screening questions using equations derived from the US clinical reappraisal sample. Although prediction accuracy was considered excellent, no other WMH country directly calibrated IPDE diagnoses. Furthermore, empirical studies about the three-cluster model of PDs have shown mixed results [61]. Some concern is also raised by the cross-sectional nature of our data, which precludes any conclusion about direction of associations or causality.

Further prospective studies with larger samples of PDs individuals are required to better understand their comorbidity with alcohol use patterns and AUD. The extensive literature on comorbidities raises the hypothesis that current diagnostic classification systems are not limited and possibly there is an overlap of symptoms allocated in different categories, which would explain part of the comorbidity between PDs and AUDs. Much has been discussed about the best system to classify psychopathology, whether categorical or dimensional [25, 62]. Many arguments exist in favor of a dimensional approach, and DSM-5 has introduced a hybrid categorical-dimensional alternative model for personality disorders [3]. The future publication of ICD-11 is promising since it proposes the reclassification of the PDs according to five domains or dimensions of personality and to levels of severity [63]. Another rapidly evolving initiative that can contribute to the understanding of the comorbidity of PDs and AUDs is the Hierarchical Taxonomy of Psychopathology (HiTOP) [25].

Assessment of possible mediators and moderators of this associations, such as drinking motives, childhood adversities and other psychiatric disorders must be included in future research. The knowledge and awareness of co-occurring mental health conditions in substance use context is crucial to improve treatment planning and to develop appropriate public policies and prevention strategies [61].

## Supporting information

S1 Data Personality and alcohol use in the SPMA. The study databank.
(XLS)

## References

[1] Global Burden of Diseases Alcohol Collaborators. Alcohol use and burden for 195 countries and territories, 1990–2016: a systematic analysis for the Global Burden of Disease Study 2016. Lancet. 2018;392(10152):1015–35. 10.1016/S0140-6736(18)31310-2 30146330 PMC6148333

[2] Silveira CM, Siu ER, Anthony JC, Saito LP, de Andrade AG, Kutschenko A, et al. Drinking patterns and alcohol use disorders in Sao Paulo, Brazil: the role of neighborhood social deprivation and socioeconomic status. PLoS One. 2014;9(10):e108355. 10.1371/journal.pone.0108355 25272008 PMC4182710

[3] American Psychiatric Association. Diagnostic and Statistical Manual of Mental Disorders, 5th Edition: DSM-5. Arlington: American Psychiatric Publishing; 2013. 991 p.

[4] Guy N, Newton-Howes G, Ford H, Williman J, Foulds J. The prevalence of comorbid alcohol use disorder in the presence of personality disorder: Systematic review and explanatory modelling. Personal Ment Health. 2018;12(3):216–28. 10.1002/pmh.1415 29611335

[5] Newton-Howes G, Foulds J. Personality disorder and treatment outcome in alcohol use disorder. Curr Opin Psychiatry. 2018;31(1):50–6. 10.1097/YCO.0000000000000375 29059106

[6] Winsper C, Bilgin A, Thompson A, Marwaha S, Chanen AM, Singh SP, et al. The prevalence of personality disorders in the community: a global systematic review and meta-analysis. Br J Psychiatry. 2019:1–10. 10.1192/bjp.2019.166 31298170

[7] American Psychiatric Association. Personality Disorders. Diagnostic and Statistical Manual of Mental Disorders, Fifth Edition. Arlington, VA: American Psychiatric Association,; 2013. p. 645–84.

[8] Trull TJ, Jahng S, Tomko RL, Wood PK, Sher KJ. Revised NESARC personality disorder diagnoses: gender, prevalence, and comorbidity with substance dependence disorders. J Pers Disord. 2010;24(4):412–26. 10.1521/pedi.2010.24.4.412 20695803 PMC3771514

[9] Casadio P, Olivoni D, Ferrari B, Pintori C, Speranza E, Bosi M, et al. Personality disorders in addiction outpatients: prevalence and effects on psychosocial functioning. Subst Abuse. 2014;8:17–24. 10.4137/SART.S13764 24701119 PMC3972129

[10] Bowden-Jones O, Iqbal MZ, Tyrer P, Seivewright N, Cooper S, Judd A, et al. Prevalence of personality disorder in alcohol and drug services and associated comorbidity. Addiction. 2004;99(10):1306–14. 10.1111/j.1360-0443.2004.00813.x 15369569

[11] Lenzenweger MF, Lane MC, Loranger AW, Kessler RC. DSM-IV personality disorders in the National Comorbidity Survey Replication. Biol Psychiatry. 2007;62(6):553–64. 10.1016/j.biopsych.2006.09.019 17217923 PMC2044500

[12] Dom G, De Wilde B, Hulstijn W, van den Brink W, Sabbe B. Decision-making deficits in alcohol-dependent patients with and without comorbid personality disorder. Alcohol Clin Exp Res. 2006;30(10):1670–7. 10.1111/j.1530-0277.2006.00202.x 17010134

[13] James LM, Taylor J. Impulsivity and negative emotionality associated with substance use problems and Cluster B personality in college students. Addict Behav. 2007;32(4):714–27. 10.1016/j.addbeh.2006.06.012 16842928

[14] Perez-Rodriguez MM, Bulbena-Cabre A, Bassir Nia A, Zipursky G, Goodman M, New AS. The Neurobiology of Borderline Personality Disorder. Psychiatr Clin North Am. 2018;41(4):633–50. 10.1016/j.psc.2018.07.012 30447729

[15] Turner S, Mota N, Bolton J, Sareen J. Self-medication with alcohol or drugs for mood and anxiety disorders: A narrative review of the epidemiological literature. Depress Anxiety. 2018;35(9):851–60. 10.1002/da.22771 29999576 PMC6175215

[16] Few LR, Grant JD, Trull TJ, Statham DJ, Martin NG, Lynskey MT, et al. Genetic variation in personality traits explains genetic overlap between borderline personality features and substance use disorders. Addiction. 2014;109(12):2118–27. 10.1111/add.12690 25041562 PMC4229407

[17] Lopez-Quintero C, Hasin DS, de Los Cobos JP, Pines A, Wang S, Grant BF, et al. Probability and predictors of remission from life-time nicotine, alcohol, cannabis or cocaine dependence: results from the National Epidemiologic Survey on Alcohol and Related Conditions. Addiction. 2011;106(3):657–69. 10.1111/j.1360-0443.2010.03194.x 21077975 PMC3227547

[18] Kessler RC. The epidemiology of dual diagnosis. Biol Psychiatry. 2004;56(10):730–7. 10.1016/j.biopsych.2004.06.034 15556117

[19] Sharp C, Vanwoerden S, Wall K. Adolescence as a Sensitive Period for the Development of Personality Disorder. Psychiatr Clin North Am. 2018;41(4):669–83. 10.1016/j.psc.2018.07.004 30447731

[20] Belcher AM, Volkow ND, Moeller FG, Ferre S. Personality traits and vulnerability or resilience to substance use disorders. Trends Cogn Sci. 2014;18(4):211–7. 10.1016/j.tics.2014.01.010 24612993 PMC3972619

[21] McAdams DP, Olson BD. Personality development: continuity and change over the life course. Annu Rev Psychol. 2010;61:517–42. 10.1146/annurev.psych.093008.100507 19534589

[22] Bjorkenstam E, Ekselius L, Burstrom B, Kosidou K, Bjorkenstam C. Association between childhood adversity and a diagnosis of personality disorder in young adulthood: a cohort study of 107,287 individuals in Stockholm County. Eur J Epidemiol. 2017;32(8):721–31. 10.1007/s10654-017-0264-9 28560537 PMC5591358

[23] Oberleitner LM, Smith PH, Weinberger AH, Mazure CM, McKee SA. Impact of Exposure to Childhood Maltreatment on Transitions to Alcohol Dependence in Women and Men. Child Maltreat. 2015;20(4):301–8. 10.1177/1077559515591270 26130105 PMC4868049

[24] Kotov R, Gamez W, Schmidt F, Watson D. Linking "big" personality traits to anxiety, depressive, and substance use disorders: a meta-analysis. Psychol Bull. 2010;136(5):768–821. 10.1037/a0020327 20804236

[25] Kotov R, Krueger RF, Watson D, Achenbach TM, Althoff RR, Bagby RM, et al. The Hierarchical Taxonomy of Psychopathology (HiTOP): A dimensional alternative to traditional nosologies. J Abnorm Psychol. 2017;126(4):454–77. 10.1037/abn0000258 28333488

[26] Verheul R. Co-morbidity of personality disorders in individuals with substance use disorders. Eur Psychiatry. 2001;16(5):274–82. 10.1016/s0924-9338(01)00578-8 11514129

[27] Trull TJ, Waudby CJ, Sher KJ. Alcohol, tobacco, and drug use disorders and personality disorder symptoms. Exp Clin Psychopharmacol. 2004;12(1):65–75. 10.1037/1064-1297.12.1.65 14769101

[28] Carter RR, Johnson SM, Exline JJ, Post SG, Pagano ME. Addiction and "Generation Me:" Narcissistic and Prosocial Behaviors of Adolescents with Substance Dependency Disorder in Comparison to Normative Adolescents. Alcohol Treat Q. 2012;30(2):163–78. 10.1080/07347324.2012.663286 22544995 PMC3335730

[29] Roncero C, de Miguel A, Fumero A, Abad AC, Martin R, Bethencourt JM, et al. Anxiety and Depression in Drug-Dependent Patients with Cluster C Personality Disorders. Front Psychiatry. 2018;9:19. 10.3389/fpsyt.2018.00019 29472875 PMC5810269

[30] United Nations, Department of Economic and Social Affairs, Population Division. World urbanization prospects—The 2005 revision. New York: United Nations Publication; 2006. p. 106.

[31] Paiva CEP. Distribuição da população na Região Metropolitana de São Paulo. Engenharia. 2009;596:511–9.

[32] Viana MC, Teixeira MG, Beraldi F, Bassani Ide S, Andrade LH. Sao Paulo Megacity Mental Health Survey—a population-based epidemiological study of psychiatric morbidity in the Sao Paulo metropolitan area: aims, design and field implementation. Braz J Psychiatry. 2009;31(4):375–86. 10.1590/s1516-44462009000400016 20098829

[33] Andrade LH, Wang YP, Andreoni S, Silveira CM, Alexandrino-Silva C, Siu ER, et al. Mental disorders in megacities: findings from the Sao Paulo megacity mental health survey, Brazil. PLoS One. 2012;7(2):e31879. 10.1371/journal.pone.0031879 22348135 PMC3279422

[34] World Health Organization, Harvard University. The World Mental Health Survey Initiative [Available from: https://www.hcp.med.harvard.edu/wmh/.

[35] Instituto Brasileiro de Geografia e Estatística-IBGE. Censo demográfico populacional do ano 2000. 2000.

[36] Kish L. A procedure for objective respondent selection within the household. J Am Stat Assoc. 1949;44(247):380–7.

[37] Kessler RC, Ustun TB. The World Mental Health (WMH) Survey Initiative Version of the World Health Organization (WHO) Composite International Diagnostic Interview (CIDI). Int J Methods Psychiatr Res. 2004;13(2):93–121. 10.1002/mpr.168 15297906 PMC6878592

[38] Loranger AW, Sartorius N, Andreoli A, Berger P, Buchheim P, Channabasavanna SM, et al. The International Personality Disorder Examination. The World Health Organization/Alcohol, Drug Abuse, and Mental Health Administration international pilot study of personality disorders. Arch Gen Psychiatry. 1994;51(3):215–24. 10.1001/archpsyc.1994.03950030051005 8122958

[39] Loranger AW. International Personality Disorder Examination: DSM-IV and ICD-10 interviews. PARS Psychological Assessment Resources; 1999.

[40] Huang Y, Kotov R, de Girolamo G, Preti A, Angermeyer M, Benjet C, et al. DSM-IV personality disorders in the WHO World Mental Health Surveys. Br J Psychiatry. 2009;195(1):46–53. 10.1192/bjp.bp.108.058552 19567896 PMC2705873

[41] Lenzenweger MF, Loranger AW, Korfine L, Neff C. Detecting personality disorders in a nonclinical population. Application of a 2-stage procedure for case identification. Arch Gen Psychiatry. 1997;54(4):345–51. 10.1001/archpsyc.1997.01830160073010 9107151

[42] Lenzenweger MF. Stability and change in personality disorder features: the Longitudinal Study of Personality Disorders. Arch Gen Psychiatry. 1999;56(11):1009–15. 10.1001/archpsyc.56.11.1009 10565501

[43] Silveira CM, Viana MC, Siu ER, de Andrade AG, Anthony JC, Andrade LH. Sociodemographic correlates of transitions from alcohol use to disorders and remission in the Sao Paulo megacity mental health survey, Brazil. Alcohol Alcohol. 2011;46(3):324–32. 10.1093/alcalc/agr007 21414952 PMC3080240

[44] Tuithof M, ten Have M, van den Brink W, Vollebergh W, de Graaf R. The role of conduct disorder in the association between ADHD and alcohol use (disorder). Results from the Netherlands Mental Health Survey and Incidence Study-2. Drug Alcohol Depend. 2012;123(1–3):115–21. 10.1016/j.drugalcdep.2011.10.030 22118715

[45] Castaldelli-Maia JM, Silveira CM, Siu ER, Wang YP, Milhoranca IA, Alexandrino-Silva C, et al. DSM-5 latent classes of alcohol users in a population-based sample: results from the Sao Paulo Megacity Mental Health Survey, Brazil. Drug Alcohol Depend. 2014;136:92–9. 10.1016/j.drugalcdep.2013.12.012 24440273 PMC4943751

[46] Graham JW. Missing data analysis: making it work in the real world. Annu Rev Psychol. 2009;60:549–76. 10.1146/annurev.psych.58.110405.085530 18652544

[47] Rhemtulla M, Little T. Tools of the Trade: Planned Missing Data Designs for Research in Cognitive Development. J Cogn Dev. 2012;13(4). 10.1080/15248372.2012.717340 24348099 PMC3857527

[48] Silvia PJ, Kwapil TR, Walsh MA, Myin-Germeys I. Planned missing-data designs in experience-sampling research: Monte Carlo simulations of efficient designs for assessing within-person constructs. Behav Res Methods. 2014;46(1):41–54. 10.3758/s13428-013-0353-y 23709167 PMC3781177

[49] Harel O, Mitchell EM, Perkins NJ, Cole SR, Tchetgen Tchetgen EJ, Sun B, et al. Multiple Imputation for Incomplete Data in Epidemiologic Studies. Am J Epidemiol. 2018;187(3):576–84. 10.1093/aje/kwx349 29165547 PMC5860387

[50] Rubin DB. Multiple imputation for nonresponse in surveys: John Wiley & Sons; 1987.

[51] StataCorp. Stata Statistical Software: Release 15. College Station, TX: StataCorp LLC. 2017.

[52] StataCorp. Stata Multiple Imputation Reference Manual: Release 15. College Station, TX: Stata Press. 2017.

[53] Santana GL, Coelho BM, Wang YP, Chiavegatto Filho ADP, Viana MC, Andrade LH. The epidemiology of personality disorders in the Sao Paulo Megacity general population. PLoS One. 2018;13(4):e0195581. 10.1371/journal.pone.0195581 29689051 PMC5978986

[54] Hasin DS, Stinson FS, Ogburn E, Grant BF. Prevalence, correlates, disability, and comorbidity of DSM-IV alcohol abuse and dependence in the United States: results from the National Epidemiologic Survey on Alcohol and Related Conditions. Arch Gen Psychiatry. 2007;64(7):830–42. 10.1001/archpsyc.64.7.830 17606817

[55] Shah R, Zanarini MC. Comorbidity of Borderline Personality Disorder: Current Status and Future Directions. Psychiatr Clin North Am. 2018;41(4):583–93. 10.1016/j.psc.2018.07.009 30447726

[56] Ruocco AC, Amirthavasagam S, Choi-Kain LW, McMain SF. Neural correlates of negative emotionality in borderline personality disorder: an activation-likelihood-estimation meta-analysis. Biol Psychiatry. 2013;73(2):153–60. 10.1016/j.biopsych.2012.07.014 22906520

[57] Martin CS, Steinley DL, Vergés A, Sher KJ. The proposed 2/11 symptom algorithm for DSM-5 substance-use disorders is too lenient. Psychol Med. 2011;41(9):2008–10. 10.1017/S0033291711000717 21557890 PMC4245011

[58] Bartoli F, Carra G, Crocamo C, Clerici M. From DSM-IV to DSM-5 alcohol use disorder: an overview of epidemiological data. Addict Behav. 2015;41:46–50. 10.1016/j.addbeh.2014.09.029 25305657

[59] Nichols LR, Samek DR, McConnell L. Key personality traits and alcohol use disorder symptoms in first and second year college students: detangling antecedent from consequence. Addict Behav. 2019;89:178–87. 10.1016/j.addbeh.2018.10.004 30316144

[60] Rubin DB. Multiple imputation for nonresponse in surveys: John Wiley & Sons; 1987.

[61] Girotto E, Mesas AE, de Andrade SM, Birolim MM. Psychoactive substance use by truck drivers: a systematic review. Occup Environ Med. 2014;71(1):71–6. 10.1136/oemed-2013-101452 24145953 PMC3888602

[62] Helle AC, Trull TJ, Watts AL, McDowell Y, Sher KJ. Psychiatric Comorbidity as a Function of Severity: DSM-5 Alcohol Use Disorder and HiTOP Classification of Mental Disorders. Alcohol Clin Exp Res. 2020;44(3):632–44. 10.1111/acer.14284 32125715 PMC7117865

[63] Tyrer P, Crawford M, Mulder R, Disorders ICDWGftRoCoP. Reclassifying personality disorders. Lancet. 2011;377(9780):1814–5. 10.1016/S0140-6736(10)61926-5 21353696

