# Correction: Alcohol use patterns and disorders among individuals with personality disorders in the Sao Paulo Metropolitan Area

**DOI:** 10.1371/journal.pone.0251857

**Published:** 2021-05-13

**Authors:** The PLOS ONE Staff

The seventh author’s name appears incorrectly. The correct name is: Yuan Pang Wang.

The ORCID iD is missing for the seventh author. Please see the author’s ORCID iD here:

- Author Yuan Pang Wang’s ORCID iD is: 0000-0001-7076-8312 (https://orcid.org/0000-0001-7076-8312).

The publisher apologizes for the errors.
